# PRESS (Propofol, Remifentanil, Electricity/EEG, Setup and Setting) to Start: Introducing a Total Intravenous Anaesthesia Checklist at a Large Teaching Hospital

**DOI:** 10.7759/cureus.56026

**Published:** 2024-03-12

**Authors:** William Woodward, James Carrannante, Kanika Dua

**Affiliations:** 1 Anaesthetics, St George's Hospital, London, GBR; 2 Anaesthesia, St George's Hospital, London, GBR

**Keywords:** infusion, propofol infusion, general anaesthesia, anaesthesia, safety, checklist, tci, total intravenous anesthesia (tiva)

## Abstract

Total intravenous anaesthesia (TIVA) is becoming more widely used, and as of yet there are few safety checks for the use of TIVA when compared to inhaled anaesthesia. This study aims to assess the feasibility and utility of introducing a TIVA checklist at a large teaching hospital.

Methods

A survey was sent out to all consultant and trainee anaesthetists at our hospital regarding their use of TIVA and errors in practice related to its use. A checklist was created based on common errors reported in the survey, errors described in NAP5 and our hospital’s standard operating procedure. The checklist was introduced, and another survey was distributed a month later to assess compliance and utility and to gain feedback.

Results

In the first survey, there were 39 responses. A total of 64% had seen an error with the use of TIVA. For those using TIVA 70% of the time or more, 31% had seen an error in the last three months. Twelve per cent of those who had seen errors found that the errors led to patient harm. Only 33% used a method to double-check for errors prior to commencing TIVA. In the follow-up survey, 80% of those who used the checklist had found it useful, and 30% had corrected an error while using the checklist. Eighty-seven per cent felt the checklist would prevent errors from being made. Eighty per cent of respondents said they would use the checklist in their future practice. The checklist was found to be more useful for trainees, and for those who use TIVA less often.

Discussion

The ‘PRESS to start TIVA” checklist has been shown to be a useful tool to prevent errors and a majority of anaesthetists at our hospital plan to use it going forward. Our data suggests that anaesthetists who are less experienced with TIVA benefit more from having a checklist. There was a marked increase in the number of anaesthetists who would use a checklist in the future, compared to those who used one in the initial survey. This shows that introducing a checklist is feasible and is likely to reduce errors going forward.

## Introduction

This article was presented as a poster at the Association of Anaesthetists Trainee Conference on 6-7th July 2023. Total intravenous anaesthesia (TIVA) is becoming more frequently used over time [1], but as of yet, there are few safety checks for the use of TIVA. This is especially true when compared to inhaled anaesthesia. Modern anaesthetic machines contain alarm systems to make the anaesthetist aware of high concentrations of inhaled anaesthetic as well as low concentrations [2]. Inhaled gases have only a certain range of percentages that can be delivered, for example, 0-8% for sevoflurane. The interlock mechanism prevents the anaesthetist from delivering two different inhaled anaesthetic gases at the same time. With current target-controlled infusion (TCI) pumps, any drug of any concentration (or no drug) could, in theory, be loaded into the pump and given to a patient. According to the fifth National Audit Project (NAP5), there is a higher incidence of awareness during TIVA than with inhaled anaesthesia. Twenty-two per cent of cases of awareness reported in the study occurred while using TIVA, but TIVA had only been used in 8% of total cases [3]. Drug errors are known to be very common within anaesthesia, with observational studies showing at least one error in nearly every anaesthetic given [4]. The most common errors within this study were giving the incorrect drug or the wrong dose. However, TIVA has been shown to be beneficial in many other areas, such as reducing postoperative nausea and vomiting, better overall survival after cancer surgery, quicker wake-up time compared to inhaled anaesthetics as well as reduced environmental impact and occupational exposure for staff to inhaled gases [5-8]. Checklists have been introduced successfully at other centres [9]. In this study, we aim to assess the feasibility and utility of introducing a TIVA checklist at a large teaching hospital and gain feedback from users to see whether this checklist is useful and whether it could be improved upon.

## Materials and methods

**Figure 1:**
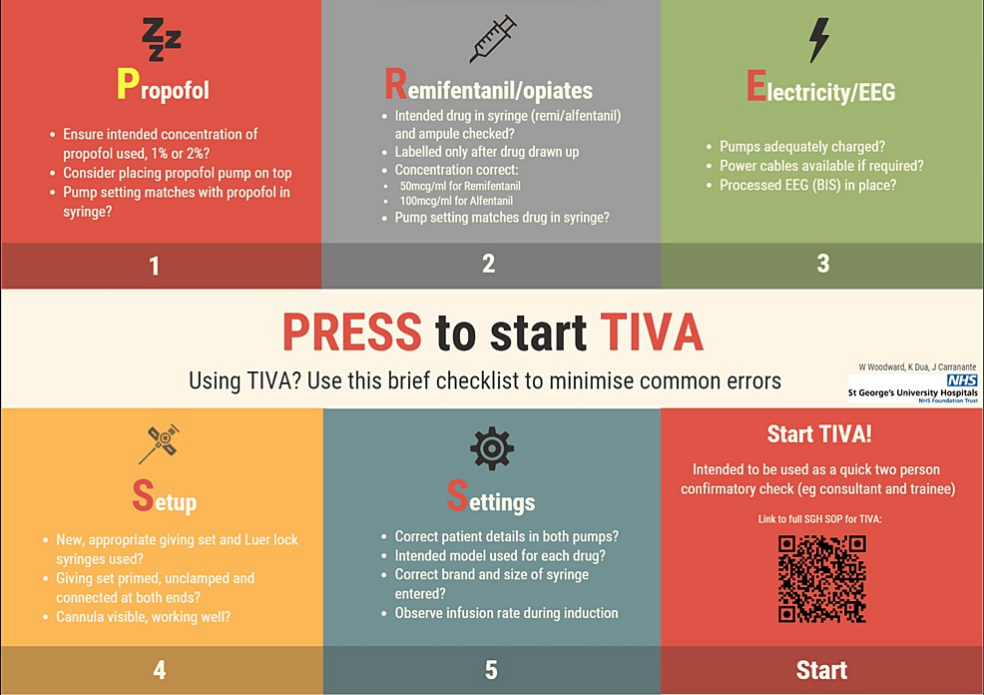
PRESS to start TIVA Checklist PRESS is an acronym for the five steps: propofol, remifentanil, electricity/EEG, setup and setting. TIVA: total intravenous anaesthesia.

To begin this study, a survey was sent out to all consultant and trainee anaesthetists at our hospital regarding their current use of TIVA, use of checklists or other methods to minimise the occurrence of errors, as well as errors they had seen in practice and whether these had led to patient harm. A checklist (Figure 1) was created based on a range of resources: common errors reported in the survey, errors described in NAP5, Association of Anaesthetists guidelines [10] and our hospital’s standard operating procedure. Checklists have been widely used in medicine worldwide and have been shown to improve compliance with standards and therefore improve patient care [11,12]. The checklist was introduced by placing posters in all adult anaesthetic rooms, on the hospital online forum and by email to all anaesthetists working at the hospital. All anaesthetists were asked to review and use the checklist while using TIVA in their routine practice. Another survey was distributed a month later to assess the compliance of using the checklist, assess its utility and gain further feedback for improvements and alterations.

The consent was obtained or waived by all participants in this study, and the study was approved by St George's Hospital Audit Department (approval AUDI003048).

## Results

**Figure 2:**
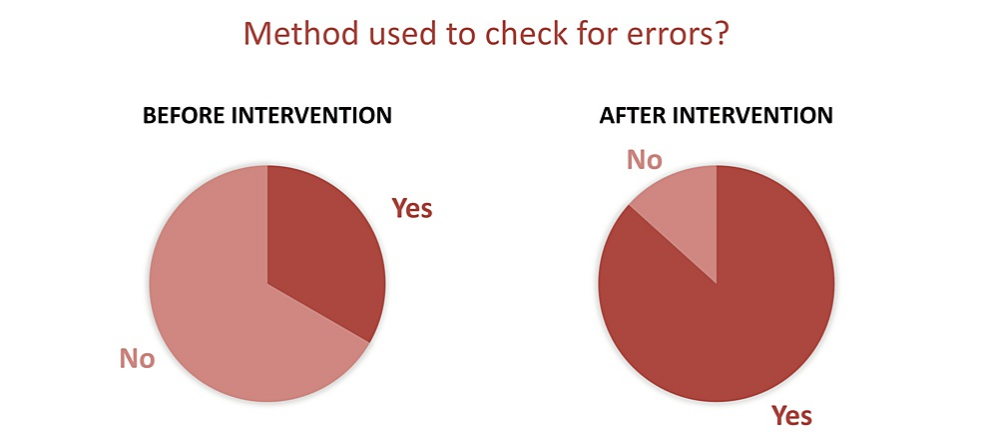
Method of double-checking comparison, before and after checklist introduction.

In the initial survey, there were 39 responses in total. Sixty-four per cent of respondents had seen an error with the use of TIVA in their practice. In those more frequent users of TIVA (using TIVA for more than 70% of the general anaesthetics they delivered), 31% had seen an error in the three months prior to responding. The most common errors reported were programming errors (11), pump failure (10), cannula failure (10), drug errors (six) and concentration errors (five). Twelve per cent of those who had seen errors found that the errors had led to patient harm. We defined patient harm as cardiovascular instability, patient awareness, or conversion to inhaled anaesthesia. Only 33% of the anaesthetists that we surveyed used a method to double-check for errors prior to commencing TIVA prior to the introduction of our checklist (Figure 2).

There were 15 respondents to the follow-up survey. Eighty per cent of those who used the checklist had found it useful, and 30% had corrected an error while using the checklist in the month since its introduction. Eighty-seven per cent felt the checklist would prevent errors from being made in their future practice while using TIVA. Eighty per cent of total respondents said they would use the checklist going forward, with 87% using some form of checklist after the intervention (Figure 2). Of the trainees surveyed, 88% reported that they would use this checklist.

## Discussion

The results suggest the checklist can be useful to anaesthetists of all grades, and it was found to be particularly useful for trainees and for those who use TIVA less often. Anaesthetists who used TIVA often in their usual practice were more likely to already have a checklist or alternative method to double-check for errors before our checklist was introduced. One of the respondents who would not use it in the future reported that this was because they already had their own checklist that they were comfortable with, showing that the vast majority of respondents used some sort of checklist after this study. This shows that when a checklist is created and available, many anaesthetists would prefer to use one to aid their technique and minimise errors. The results also show that the vast majority felt that using this checklist would reduce errors, and therefore reduce patient harm.

Plans for improving the use of the poster going forward include considering the use of QR codes in relevant and accessible areas for anaesthetists using TIVA, such as on top of the TCI pumps, with a link to the poster itself. QR codes have been used in other areas of medicine to improve the availability of checklists [13]. Checklists such as this one could be used in combination with other advances, such as continuous measurement of cannula pressure and processed electroencephalogram monitoring, to improve the overall safety of TIVA [14,15].

## Conclusions

The ‘PRESS to Start TIVA’ checklist has been shown to be a useful tool to prevent errors and a majority of anaesthetists at our hospital plan to use it going forward. Our data suggests that anaesthetists who are less experienced with TIVA benefit more from having a checklist. There was a marked increase in the number of anaesthetists who would use a checklist in their future practice, compared to those who used one prior to the introduction of this checklist. This shows that introducing a checklist at a large teaching hospital is feasible and can reduce errors. It is something that could be introduced more widely across the NHS and can standardise the use of TIVA within hospitals. These benefits, combined with other advancements in this area, can lead to reductions in patient harm such as awareness, cardiovascular instability and other negative outcomes that can be associated with TIVA use as TIVA becomes more popular over the coming years.

## References

[1] Kane AD, Soar J, Armstrong RA (2023). Patient characteristics, anaesthetic workload and techniques in the UK: An analysis from the 7th National Audit Project (NAP7) activity survey. Anaesthesia.

[2] Brattwall M, Warrén-Stomberg M, Hesselvik F, Jakobsson J (2012). Brief review: Theory and practice of minimal fresh gas flow anesthesia. Can J Anaesth.

[3] Pandit JJ, Andrade J, Bogod DG (2014). The 5th National Audit Project (NAP5) on accidental awareness during general anaesthesia: Summary of main findings and risk factors. Anaesthesia.

[4] Bratch R, Pandit JJ (2021). An integrative review of method types used in the study of medication error during anaesthesia: Implications for estimating incidence. Br J Anaesth.

[5] Jones C, Harris J (2021). Total intravenous anaesthesia. Br J Hosp Med.

[6] Yap A, Lopez-Olivo MA, Dubowitz J, Hiller J, Riedel B (2019). Anesthetic technique and cancer outcomes: A meta-analysis of total intravenous versus volatile anesthesia. Can J Anaesth.

[7] Varughese S, Ahmed R (2021). Environmental and occupational considerations of anesthesia: A narrative review and update. Anesth Analg.

[8] Irwin MG, Trinh T, Yao CL (2009). Occupational exposure to anaesthetic gases: A role for TIVA. Expert Opin Drug Saf.

[9] Fleming RJ (2020). PERUSE before you infuse. Anaesthesia News.

[10] Nimmo AF, Absalom AR, Bagshaw O (2019). Guidelines for the safe practice of total intravenous anaesthesia (TIVA): Joint guidelines from the Association of Anaesthetists and the Society for Intravenous Anaesthesia. Anaesthesia.

[11] W-Dahl A, Robertsson O, Stefánsdóttir A, Gustafson P, Lidgren L (2011). Timing of preoperative antibiotics for knee arthroplasties: Improving the routines in Sweden. Patient Saf Surg.

[12] Takala RS, Pauniaho SL, Kotkansalo A (2011). A pilot study of the implementation of WHO surgical checklist in Finland: Improvements in activities and communication. Acta Anaesthesiol Scand.

[13] Dixon JL, Smythe WR, Momsen LS, Jupiter D, Papaconstantinou HT (2013). Quick response codes for surgical safety: A prospective pilot study. J Surg Res.

[14] McGuire A, Broome I (2017). Improving TIVA safety through measurement of peripheral venous pressure. Anaesthesia.

[15] Hajat Z, Ahmad N, Andrzejowski J (2017). The role and limitations of EEG-based depth of anaesthesia monitoring in theatres and intensive care. Anaesthesia.

